# Loop cautery for chronic lymphangioma removal

**DOI:** 10.1016/j.jdcr.2025.01.044

**Published:** 2025-03-25

**Authors:** Riyad N.H. Seervai, Lynna J. Yang, Elizabeth G. Berry

**Affiliations:** a Department of Dermatology, Oregon Health & Science University, Portland, Oregon; b Department of Dermatology, Northwestern University Feinberg School of Medicine, Chicago, Illinois

**Keywords:** chronic lymphedema, electrosurgery, loop cautery, lymphangioma, metastatic melanoma, supportive oncodermatology, survivorship

## Introduction

Lymphangiomas are benign vascular malformations that can occur on the skin or mucous membranes, appearing either congenitally or as an acquired complication of chronic lymphedema following surgery, malignancy, or trauma.^1^ Lymphangiomas can significantly impact patients’ quality of life due to pain, pruritus, and increased infection risk.^1^ Surgical excision is the traditional treatment of choice but has a 25% to 50% recurrence rate within the first 3 months.^2^ For patients who are not surgical candidates, alternative treatment options include electrodesiccation, electrofulguration, radiofrequency ablation, and carbon dioxide laser, although these treatments are thought to be more for symptomatic relief than curative.^2^ As such, treatment options for chronic lymphangiomas are limited or ineffective, and adequate management of these burdensome lesions represents an unmet need. Here, we present a novel approach using electrosection with loop cautery to treat extensive, painful lymphangiomas on the scrotum and penis of a patient with severe chronic scrotal lymphedema following bilateral inguinal lymph node dissection for metastatic melanoma.

## Case presentation

The patient is a 44-year-old man with a history of BRAF^V600K^-positive melanoma of the left leg, originally diagnosed 16 years ago as stage IIIB with 2.7 mm Breslow depth and positive sentinel lymph nodes on biopsy. His family history is significant for mucosal melanoma (mother) and pancreatic adenocarcinoma (father), both of whom died from progression of their disease. His clinical course was initially complicated by inguinal lymph node metastases, requiring extensive bilateral lymph node dissection, and additional metastases to the back, flank, abdominal wall, and small bowel. He had multiple recurrences requiring excisions, systemic treatments (temozolomide, carboplatin, and trametinib), and radiation. He had a robust response to vemurafenib and is without evidence of disease for almost 10 years.

**Fig 1:**
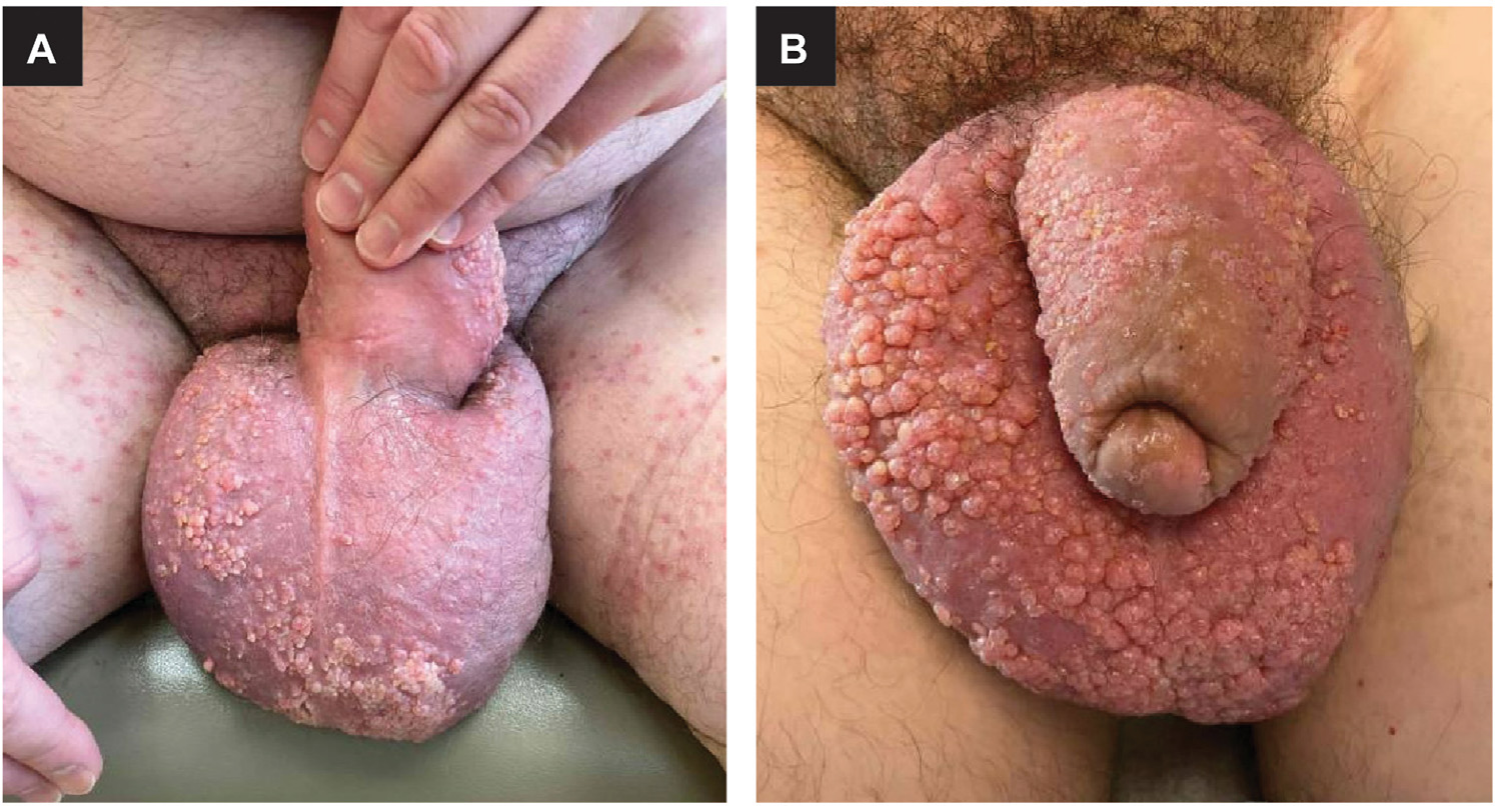
Progression of lymphangiomas before loop cautery treatment. **A,** Initial presentation with dozens of *pink* vascular papules scattered on the scrotum with some areas of confluent plaques. **B,** Ten months later, there are hundreds of larger, confluent lymphangiomas over the scrotum and penile shaft.

As a result of his extensive bilateral inguinal lymph node dissections, the patient developed severe chronic lymphedema of the lower extremities, perineum, scrotum, and penis. Over time, multiple lymphangiomas arose on the scrotum and penis. The patient experienced recurrent cellulitis, ultimately requiring daily antibiotic prophylaxis. Attempts to remove the lymphangiomas with electrodessication were unsuccessful. At the time he presented to our clinic 3 years ago, an exam showed dozens of pink vascular papules scattered across a severely edematous scrotum and penis (Fig 1, *A*). The lymphangiomas continued to spread, with hundreds to thousands of papules and vesicles in confluent sheets covering his scrotum, penis, perineum, and mons pubis (Fig 1, *B*). Initial efforts at electrodessication continued unsuccessfully; the patient experienced intolerable pain requiring continuous topical anesthesia and reported attempts to remove the papules and vesicles at home with a nail clipper.

**Fig 2:**
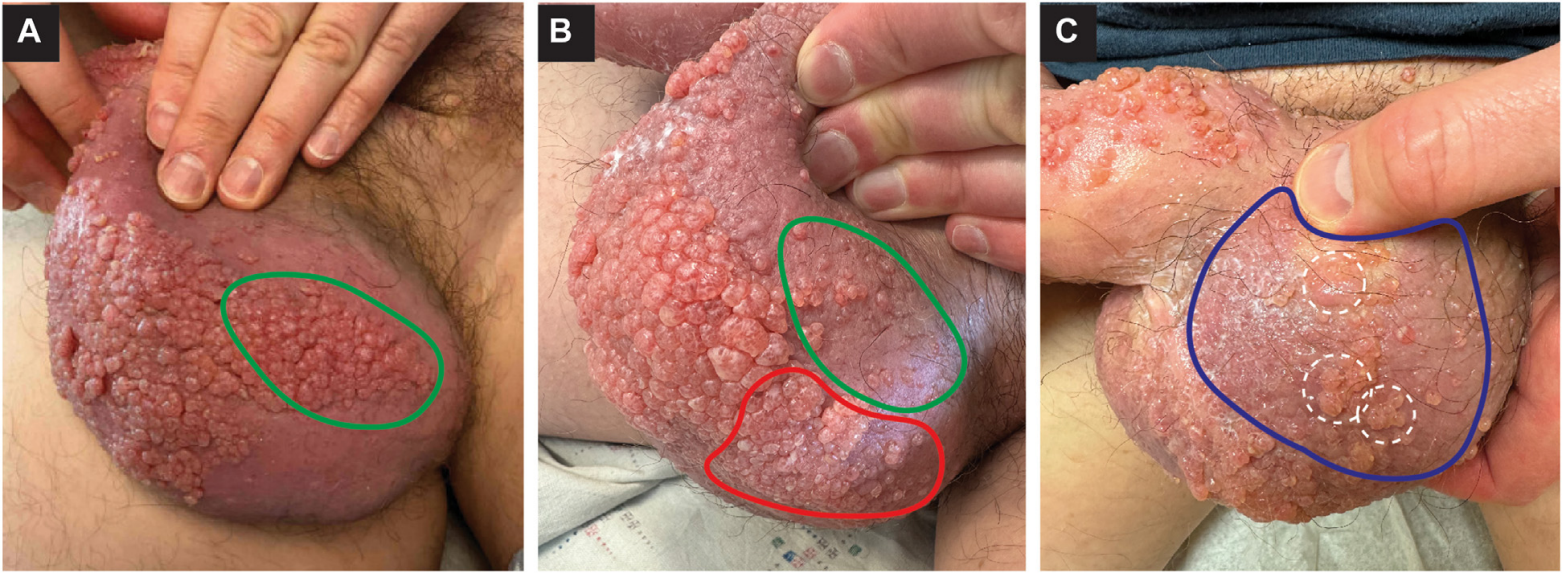
Improvement in lymphangiomas over 2 sessions of loop cautery treatment. **A,** Confluent sheets of *pink* vascular papules on the left scrotum. The area in *green* was treated during the visit. **B,** Two months later, there is relatively low recurrence in the treated area (circled in *green*) but progression at untreated sites (*red*). **C,** Five weeks later, the collectively treated areas (circled in *blue*) show minimal recurrence or scarring. The areas circled in *white* were so thick that they were only thinned with the treatment, but the patient reported decreased pain in these areas as well.

**Fig 3:**
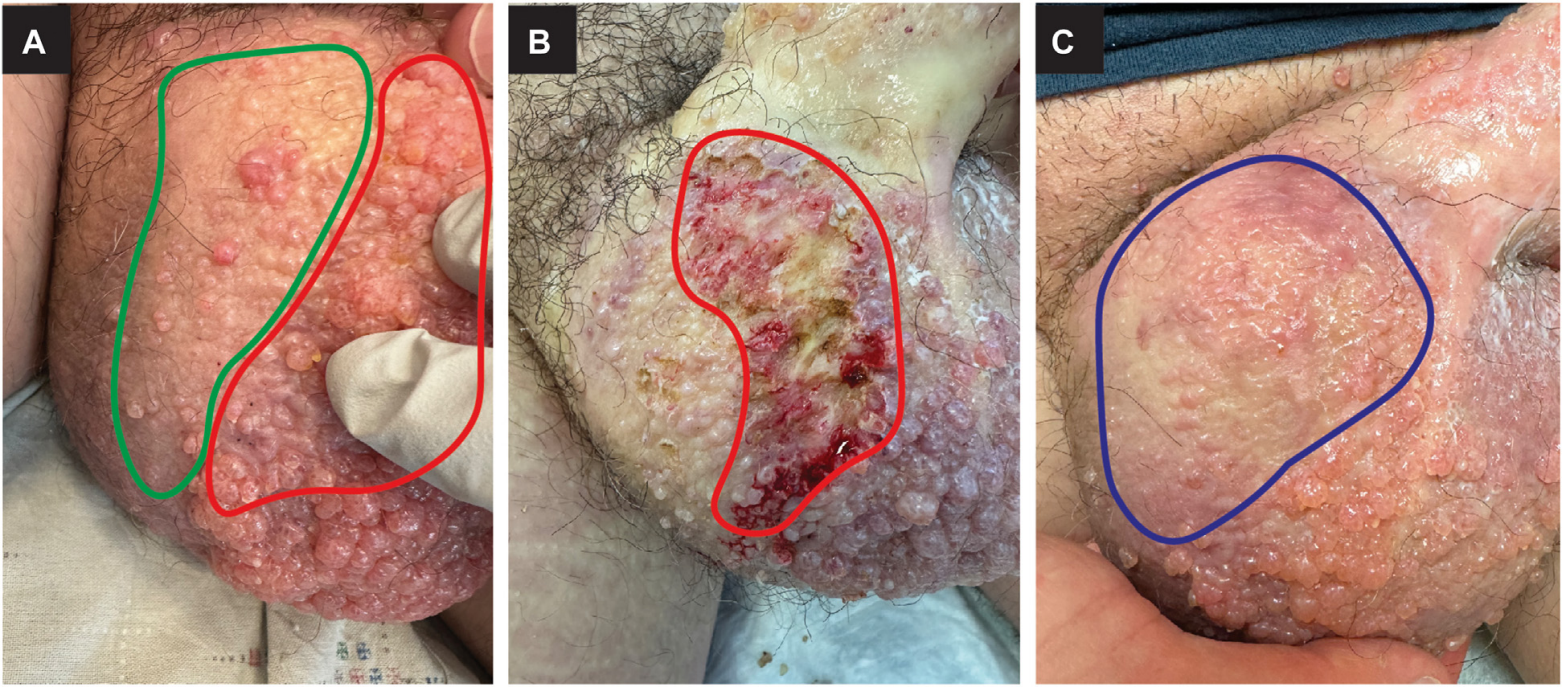
Clinical course before, during, and after loop cautery treatment. **A,** Confluent sheets of pink vascular papules on the right scrotum and penile shaft. The patient requested treatment of the general area indicated in *red*. The area circled in *green* was treated at an appointment 2 months before. **B,** The requested area (circled in *red*) was treated with loop cautery. **C,** Five weeks later, the treated areas (circled in *blue*) are healed with minimal recurrence or scarring.

We attempted electrosection with loop cautery to remove the patient’s rapidly proliferating lymphangiomas, with optimal settings identified through careful titration (Supplementary Video, available via Mendeley at https://data.mendeley.com/datasets/yz6nxw2z55/1). At each visit, his most painful or bothersome areas were pretreated with lidocaine 2.5%/prilocaine 2.5% topical cream an hour before the procedure. Additional local anesthesia was obtained with injection of 1% lidocaine buffered with 1:100,000 epinephrine directly into the affected areas. The lymphangiomas were then removed by loop cautery, with minimal bleeding and a low recurrence rate at the treated areas (Figs 2 and 3). The patient’s office procedures are billed under Current Procedural Terminology code 17111 (destruction ≥15 benign lesions) and have been covered by his medical insurance plan.

## Discussion

Here, we report the safe and successful management of lymphangiomas on the penis and scrotum using electrosection with loop cautery. This approach offers a promising alternative to existing treatments, demonstrating minimal pain, low recurrence rates, and high patient satisfaction. The decision to pursue this treatment modality was multifactorial. While surgical management with excision and repair using flaps or grafts is generally considered the gold standard,^3^, ^4^, ^5^ the high recurrence rate (25% to 50%) and significant morbidity associated with the surgery led our urology team to recommend exploring local therapy options first. Carbon dioxide laser, reported to be successful with minimal scarring in some cases,^6^ was deemed unsuitable for our patient due to the widespread nature of his lymphangiomas and the associated pain.

The electrosection technique in our procedure uses high amperage and low voltage current with a loop electrode to cut tissue with quick, precise excision and rapid hemostasis. Electrosection is not supported by the Hyfrecator system and requires specialized electrosurgical units. Other reports have suggested radiofrequency ablation, electrodessication, and electrocoagulation as treatment modalities for lymphangiomas.^7^, ^8^, ^9^ The key distinction between our approach and other electrosurgical methods lies in the mechanism of action and tissue interaction.^10^ Radiofrequency ablation uses very high-frequency electrical current to heat and destroy tissue. Electrodessication uses low amperage, high voltage electrical pulses through a single point to dehydrate superficial tissue. Electrocoagulation utilizes high amperage and low voltage current for deeper tissue destruction.^10^ None of these techniques allow for excision as electrosection with loop cautery does.

One of the primary concerns with using loop cautery for lymphangioma treatment is infection, particularly Fournier’s gangrene. This was a concern in our patient with a history of recurrent cellulitis. However, the patient’s attempts to self-remove his lymphangiomas at home presented an even greater infection risk. This led to a shared decision-making process where the potential benefits of controlled treatment with loop cautery were deemed to outweigh the risks of infection. With monthly scheduled procedure visits, removing an increasing number of lymphangiomas each time, the patient has stopped trying to remove them at home. Other complications of the procedure include pain and bleeding. In our patient, pain was effectively managed using a combination of topical and local anesthesia prior to treatment. Most recently, our patient underwent a bilateral genitofemoral nerve block that has greatly improved his symptoms at baseline and during the lymphangioma removals. Bleeding is minimal during the electrosection procedure and is easily managed (Supplementary Video, available via Mendeley at https://data.mendeley.com/datasets/yz6nxw2z55/1).

With continued advances in cancer treatments, patients are living significantly longer. In this new age of cancer management, it is critical to address the long-term side effects of these anticancer therapies. Lymphedema and associated lymphangiomas are significant quality-of-life issues for many cancer survivors who have undergone lymph node dissections. We strongly believe electrosection with loop cautery offers a safe solution to managing the challenging complication of lymphangiomas in caring for cancer survivors.

While this case focuses on lymphangiomas in the setting of cancer survivorship, the loop cautery treatment approach can potentially be applied to acquired lymphangiomas from other etiologies as well as lymphangioma circumscriptum. Unlike acquired lymphangiomas, lymphangioma circumscriptum have rare reports of transformation into squamous cell carcinoma, lymphosarcoma, or angiosarcoma.^8^ In such cases, the lymphangiomas can be removed prophylactically with loop cautery to prevent malignant transformation.

In conclusion, this case demonstrates a novel and promising approach to treating symptomatic lymphangiomas with electrosection via loop cautery. While the results are encouraging, further studies are needed to understand long-term outcomes, refine the technique, and establish its efficacy and safety profile in a larger patient population. Additionally, future research should focus on developing preventive strategies and early interventions to mitigate the development of severe lymphedema and associated lymphangiomas in high-risk patients, such as those undergoing lymph node dissections for cancer treatment.

## Conflicts of interest

Dr Seervai is a member of the Derm In-Review Advisory Council (DIRAC) affiliated with SanovaWorks. Dr Berry serves as a consultant for Bristol Myers Squibb. Dr Yang has no conflicts of interest to declare.
